# Adsorption of phenol and methylene blue contaminants onto high-performance catalytic activated carbon from biomass residues

**DOI:** 10.1016/j.heliyon.2024.e41150

**Published:** 2024-12-12

**Authors:** Numfor Linda Bih, Mwemezi J. Rwiza, Asha S. Ripanda, Assia Aboubakar Mahamat, Revocatus L. Machunda, Joon Weon Choi

**Affiliations:** a School of Materials, Energy, Water and Environmental Sciences (MEWES), The Nelson Mandela African Institution of Science and Technology (NM-AIST), School of Materials, Energy, Water and Environmental Sciences (MEWES), P.O. Box, 447, Arusha, Tanzania; b Graduate School of International Agricultural Technology, Department of Green Eco System, Engineering, Seoul National University, Pyeongchang, 25354, Gangwon-do, South Korea; c Nile University of Nigeria: Abuja, Federal Capital Territory, Airport Rd, Jabi, 900001, Abuja, Nigeria

**Keywords:** Adsorption, Activated carbon, Methylene blue, Phenol, Kinetic model, Isotherm model

## Abstract

Organic contaminants from wastewater toxicity to the environment has increased during the last few decades and, therefore, there is an urgent need to decontaminate wastewater prior to disposal. This study aimed to create a high surface area catalytic activated carbon (AC) under same carbonization conditions for phenol and methylene blue (organic wastewater) decontamination. *Moringa oleifera* husk (MH), sesame husk (SH), and baobab husk (BH) were used to prepare activated carbon for the removal of methylene blue (MB) and phenol (Ph). After characterization of the adsorbent, the BET surface areas of the *M. oleifera* husk activated carbon (MHC), sesame husk activated carbon (SHC), and baobab husk activated carbon (BHC) were 1902.30 m^2^/g, 1115.90 m^2^/g, and 1412.40 m^2^/g, respectively. Mono-adsorption and binary-adsorption systems were studied for Ph and MB adsorption. Furthermore, the effect of initial organic waste concentration, contact time, pH, temperature and AC dosage, on adsorption capacity were studied. The mono adsorption system isotherms and kinetics studies used to analyze Phenol and MB adsorption best fitted Langmuir and pseudo-second-order models. The Freundlich isotherm and pseudo-second-order model best fitted the experimental data for the binary-adsorption system. The high maximum adsorption capacities of organic waste for the single and binary systems were 352.25–855.96 mg/g and 348.90–456.39 mg/g, respectively. The results showed that the high surface activated carbon produced had the potential to adsorb high concentrations of MB and Phenol contaminants.

## Graphical abstract

**Figure:**
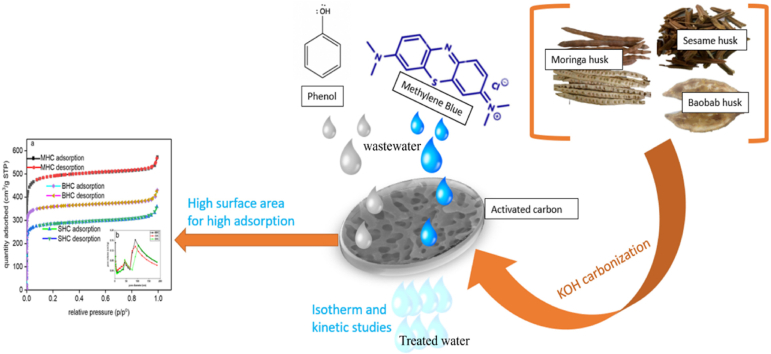

## 1 Introduction

The use of synthetic methylene blue (MB) in the textile industry has increased significantly in recent years. Due to their harmful effects on living organisms, MB and phenol present in industrial effluents are a fundamental problem. In general, just 8 % of this type of wastewater is treated before being disposed of [1], and this source typically accounts for 80 % of the overall emissions generated by the textile industry [2,3] and causes the largest amount of pollution [4]. Current methodologies such as chemical precipitation, nano-filtration and reverse osmosis are used for wastewater treatment but are very costly. Therefore, it is necessary to use a low-cost technique such as adsorption that can utilize biomass residue activated carbon as adsorbents for wastewater treatment.

Since cationic MB is used in many different industries including plastics, paper, cosmetics, textiles, inks, food, and pharmaceuticals, annual global production is around 2 million tons [5,6]. Given its relative stability as a chemical and difficulty in being degraded in wastewater treatment facilities using physical, chemical, or biological treatments, MB is one of the substances of greatest concern [7,8]. Phenol (Ph) is a type of aromatic chemical compound used in the production of compounds like phenolic resins, bisphenol A, and adipic acid. The global phenol (Ph) market volume in 2022 was about 11.80 million metric tons and the market volume is expected to increase to about 14.53 million metric tons by 2030 [9]. Due to their impact on human cells, phenols are one of the pollutants that are very hazardous and can lead to blurred vision, oral irritation, and diarrhea [10,11]. Environmental MB and phenol discharge is a problem since it raises toxicological and aesthetic issues [12]. Therefore, it is necessary to eliminate them from wastewater before the industrial wastewater is discharged. Phenols and MB are widely used chemicals and, consequently, these pollutants may be found in the wastewater of many industries generating considerable amounts of phenolic and colored wastewater [13] that is toxic and even carcinogenic [14], posing a serious hazard to aquatic living organisms [6].

Biomass husks are low-cost material produced by agricultural operations and usually disposed of as landfill waste. Agricultural byproducts and residues have been extensively identified as good sources of activated carbon [6,15]. The production of activated carbon can enhance a country's economic and environmental sustainability. Therefore, producing activated carbon (AC) from moringa, sesame, and baobab husks is an excellent waste management practice and an economic value addition. Biomass waste-activated carbon has been shown to have high adsorption capacity of organic contaminants [16,17]. However, current research has focused on single heavy metal ion adsorption or binary heavy metal ion adsorption, with only a few studies comparing single organic adsorption in the presence of binary organic adsorbates. The properties of AC are heavily influenced by the precursor material, catalytic agent, surface area, pore volume, and production process [[18], [19], [20]]. A variety of biomass residues, such as shells, husks or pods, tree bark, seeds, chicken bone, leaves, flowers, or stones, can be used to produce AC [[20], [21], [22], [23], [24], [25]]. Activated carbon from biomass waste is carbon-rich, inexpensive, and has a highly active surface.

The raw material (precursor) and the activation agent are the two most critical elements influencing the cost of AC through chemical activation (catalytic agents, such as NaOH, ZnCl_2_, KOH, etc.) [[26], [27], [28]], physical activation (steam, air, or CO_2_) [29], or when both techniques are applied [30]. Chemical activation entails impregnating the raw material with a catalytic agent usually under N_2_ gas and then carbonizing the impregnated precursor at the prescribed temperature. The activation of biomass waste with potassium hydroxide (KOH) is frequently employed in the chemical activation process hence, high surface area AC is produced without surface modification. According to literature, alkaline hydroxides such as KOH promotes high surface area, high -OH functional group on the surface of the activated carbon and environmentally friendly synthesis [31,32] hence, KOH is frequently chosen over other catalytic agent for the generation of AC [[33], [34], [35]]. Due to potassium intercalation within carbon lattices and carbon oxidation, KOH activation produces an important quantity of micropores activated carbon and extensive functional sites on the surface [36]. There has been increasing research interest in the activation of carbon with a significant amount of micropores generated from biomass using KOH as the activation agent [37,38].

To the best of our understanding, no study has been conducted on utilization of KOH as catalytic agent for *Moringa oleifera* husk, Sesame husk and baobab husk (biomass residue) for mono and binary adsorption of methylene blue and phenol. The utilization of activated carbon produced under the same condition for adsorption of contaminant is crucial in the determination of presenting data and scientific research for commercializing AC and application in industries. In this study, accessible and cost-effective catalytic KOH-activated carbons were synthesized, and their potential and utility for the adsorption of MB and phenol were demonstrated. The effects of MB and Ph concentration, AC dosage and contact time, effect of pH and the adsorption removal percentage were initially investigated. Furthermore, kinetic, isotherm, and thermodynamic models were studied. The catalytic activated carbon adsorbent was demonstrated to be extremely promising for organic adsorption applications at high concentrations of MB and Ph. For the adsorption of methylene blue and phenol aqueous solutions, the chemically generated activated material was shown to have substantial potential application as an adsorbent material. The adsorption of phenol and methylene blue as target contaminants from mono and binary polluted solutions on *Moringa oleifera*, sesame and baobab husks activated carbon is being reported for the first time in this study.

## 2 Materials and methods

### 2.1 Materials

*Moringa oleifera* was collected from the NM-AIST campus in Arusha, sesame husk from Babati, and baobab from Dodoma Tanzania. Sodium hydroxide (97 % NaOH, Sigma Aldrich) and hydrochloric acid (36 % HCl, ReAgent) were used to balance the pH of the synthetic contaminants: methylene blue (97 % MB, Sigma Aldrich) and phenol (99 %, Sigma Aldrich). Potassium hydroxide (99.99 % KOH, Sigma Aldrich) was used as the catalytic agent for activation. All chemicals used for experiments were procured from trusted manufacturers and were of analytical grade.

### 2.2 Adsorbent synthesis

The biomass waste was washed, oven-dried at 105 °C for 48 h, and sieved to 1 mm. The sieved samples were immersed in a KOH ratio of 1:2 for 24 h and dried for 24 h at 105 °C before carbonization at 700 °C for 1 h under N_2_ gas [39]. The activated biomass was then washed with 250 mL 0.1 M HCl and distilled water until a pH of 6–7 was achieved. The samples were oven-dried for 24 h at 105 °C, crushed in a mortar and pestle, and then stored for further use in a desiccator. The samples were labeled *Moringa. oleifera* husk activated carbon (MHC), sesame husk activated carbon (SHC), and baobab husk activated carbon (BHC).

### 2.3 Characterization of the activated carbon and instrumentation

Brunauer, Emmett and Teller (BET), Fourier Transform Infrared (FT-IR) spectroscopy, Field Emission Scanning Electron Microscopy (FE-SEM) with an elemental analyzer (EDS), and thermogravimetric analysis (TGA) were used to characterize the adsorbents. The concentrations of the methylene blue and phenol were obtained by absorbance measurements using a beam UV–vis spectrophotometer (Genesys 180, Thermo Scientific) at wavelengths of 668 and 207 nm, respectively. An incubator shaker was used at 30 °C for the adsorption experiment. In this investigation, a field-emission scanning electron microscope (SEM, SIGMA, Carl Zeiss, UK) was used to assess the surface morphology of the adsorbents, and a high-resolution FT-IR spectrophotometer (FTIR, Vertex-80V/Hyperion2000, Germany) was used to obtain the chemical functional groups. Information on the N_2_ adsorption-desorption isotherms was gathered using the Brunauer-Emmett-Teller method (BELSORP-max, Microtrac BEL Corp, Japan). A thermogravimetric analyzer (Mettler-Toledo, AG, Switzerland) was used to assess the thermal stability of the AC in the range of 25–800 °C at a heating rate of 10 °C min^−1^ in a nitrogen environment at 50 mL min^−1^ flow. The elemental compositions of MHC, SHC, and BHC were analyzed using a PerkinElmer elemental analyzer.

### 2.4 Batch adsorption tests

Methylene blue and phenol were used as adsorbates to study the adsorption behavior of the activated carbon samples. Stock solutions of methylene blue (1000 mg/L) and phenol (1000 mg/L) were prepared and diluted to 50–600 mg/L. An adsorption test determined that the adsorbate volume and adsorbent mass were 100 mL and 0.5 g/L, respectively. The samples were then placed in an incubator shaker at 30 °C for 120 min at a pH of 6–7. Furthermore, varied concentrations and contact times with a fixed amount of adsorbent of 0.5 g/L of AC in 250 mL Erlenmeyer flasks were studied for binary adsorption. The solid/liquid mixture was then filtered using PTFE-membrane filters (0.45 μm pore size) after the adsorption test. The residual adsorbate in the solution was examined using the UV–vis analysis technique. MB and phenol have maximum wavelengths of 668 and 270 nm, respectively. The calibration curves employed concentration ranges of 1–8 ppm for MB and 10–100 ppm for phenol. Furthermore, the correlation coefficient (R^2^) was employed to assess the fit quality of each curve and maximum adsorption capacity of the adsorbent. Adsorption of phenol on the adsorbents was denoted as PhMHC, PhSHC, and PhBHC, and adsorption of methylene blue was denoted as MBMHC, MBHC, and MBBHC. Thermodynamic models were also studied at temperature 20–40 °C.

The removal efficiency (%R) of MB and phenol, and the amount of contaminants adsorbed (q_e_, mg/g) were computed using Eq. (1) and (2), respectively, as follows:

(1) qe=V(c0−c)m

(2) %R=(c0−c)c0×100

where q_e_ is the adsorption capacity (mg/g), c_0_ and c are the initial and final MB concentrations (mg/L), respectively, V is the volume (mL), and m is the mass (g) of the adsorbent.

The effects of the initial concentrations of MB and phenol on the AC adsorption in 100 mL aqueous solution of the single-organic system were studied at various MB and phenol concentrations ranging from 50 to 600 mg/L at 120 min. Similarly, a 100 mL mixture of MB and phenol solutions each with a concentration of 300 mg/L were used for Ph-MB binary system adsorption, similar to the single adsorption system, at a temperature of 30 °C, 150 rpm, 120 min, and an optimum dosage of 0.5 g/L. These experiments were conducted the same for each adsorbent: MHC, SHC, and BHC.

To investigate the effects of contact time, Erlenmeyer flasks containing 100 mL at an initial concentration of 300 mg/L of MB and phenol were mixed with a 50 mg adsorbent dosage. The Erlenmeyer flasks were then shaken at 150 rpm for between 1 and 480 min contact time. Each adsorption solution was collected with a 25 mm syringe and a 0.45 μm syringe filter of PTFE-H (polytetrafluoroethylene).

The adsorbent dosages for adsorption were varied between 0.1 and 2 g/L. A concentration of 300 mg/L each of MB and phenol solution was used, and the binary mixture was (1:1). The samples were filtered after adsorption for UV-spectrometer analysis to determine the concentrations of MB and phenol adsorbed.

The AC samples' point of zero charge (pH_PZC_) was determined using the mass titration (MT) method, which was first proposed by Noh and Schwarz [40]. The pH value at which the surface net charges of the AC are zero was found using the point of zero charge (pHpzc). At pH values greater than pHpzc, AC surface is negatively charged which encourages the adsorption of cationic compounds. On the other hand, at pH values less than pHpzc, the AC surface is positively charged which can repel the cations [41].

## 3 Results and discussion

### 3.1 Characterization of the adsorbents

#### 3.1.1 Fourier transform infra-red spectroscopy (FT-IR)

**Fig. 1:**
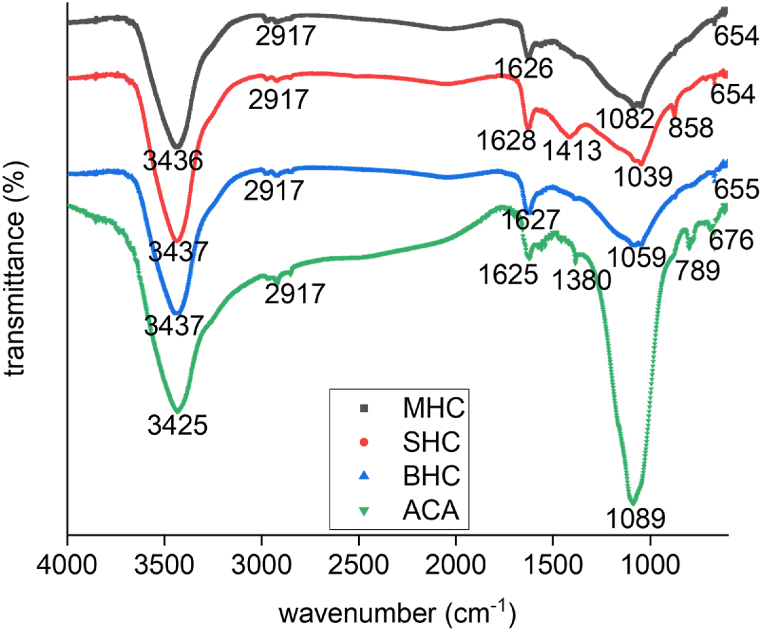
FTIR spectra of activated carbon (MHC, BHC, SHC) and activated carbon after adsorption (ACA).

The surface structure of carbon-oxygen (functional groups) and the surface behavior of adsorbents have a considerable influence on MB and phenol adsorption capacity. Functional groups for the MHC, BHC, and SHC samples were determined using Fourier-transform infrared (FT-IR) spectroscopy. The occurrence of FT-IR spectra with different peak frequences, relates to energy changes of functional group. Therefore, after adsorption, formation of new peaks or disappearance of peaks during adsorption, were observed on FT-IR spectra. Before adsorption and after adsorption FT-IR analysis of contaminant was shown in Fig. 1.

The peaks of MHC, BHC, SHC and activated carbon after adsorption (ACA) at 3436 cm^−1^, 3437 cm^−1^, 3437 cm^−1^, and 3425 cm^−1^ respectively, corresponded to the -OH groups of carboxylic acids and phenolic groups. Stretching and aliphatic group vibrations of C=O at 1626 cm^−1^, 1627 cm^−1^, and 1628 cm^−1^, and C-O at 1082 cm^−1^ and 1050 cm^−1^ in the ionic carboxylic groups (-COO-), peaks were observed for MHC, BHC, and SHC, respectively. Similarly, a CH- in-plane deformation was responsible for vibration at 64 cm^−1^ (MHC and SHC), 665 cm^−1^ (BHC), 858 cm^−1^ (SHC) and 676 cm^−1^ (ACA). After adsorption, identical peaks at different locations and increase in intensities were seen on ACA. The peaks at 3436 cm^−1^, shifted to 3425 cm^−1^, indicating active involvement of contaminants in the adsorption process as also illustrated in a study by Albert et al. [42] and Allahkarami et al. [43]. A new peak 1380 cm^−1^ is attributed to N-O bond after adsorption which was absent before adsorption. The new broad band from 676 cm^−1^ to 789 cm^−1^ was ascribed to C–Br (from KBr during sample preparation) stretching and suggest the presence of MB [44,45].

The spectral analysis at 1089 cm^−1^ after adsorption demonstrated that most of the adsorption process involved bonded –OH groups and C=O stretching giving it a high intensity strong dipole bond (O-H) between activated carbon and organic contaminant [46].

#### 3.1.2 Surface morphology

**Fig. 2:**
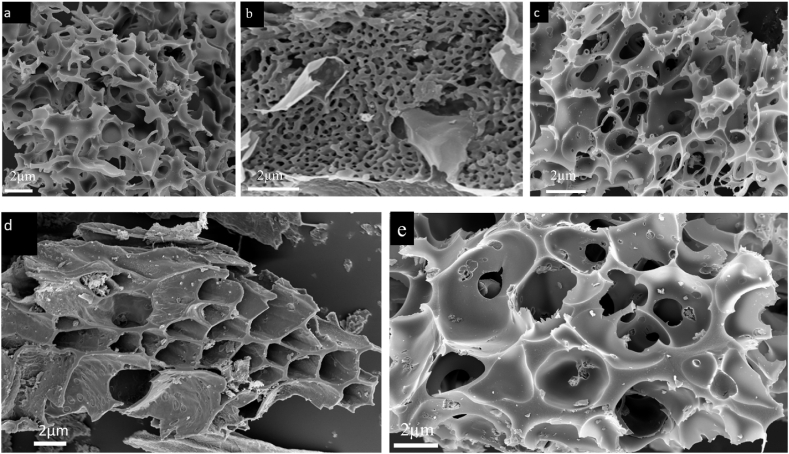
SEM micrographs of activated carbon before adsorption (a) MHC, (b) BHC, (c) SHC, and after adsorption (d) AC.

**Fig. 3:**
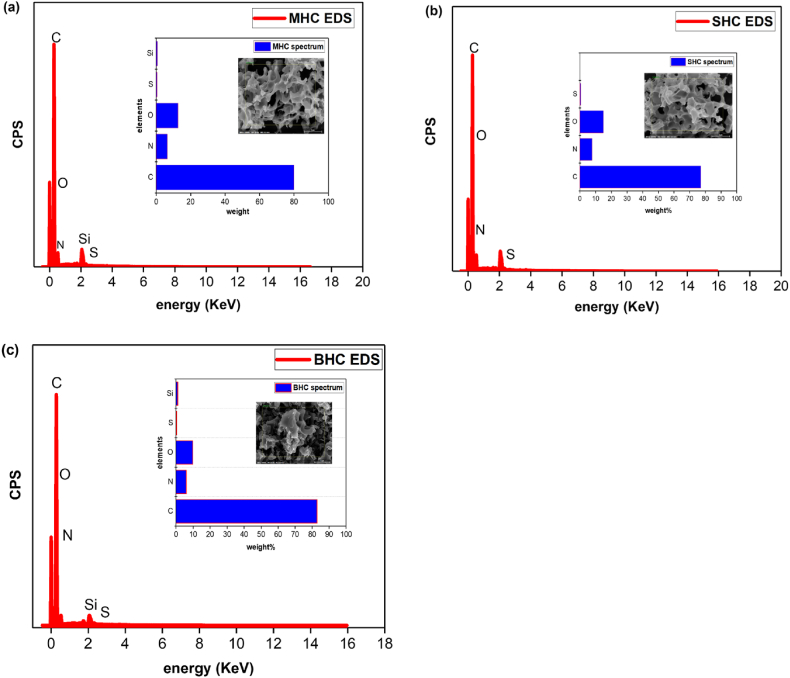
EDS composition of activated carbon (a) MHC, (b) BHC, and (c) SHC.

Scanning electron microscopy (FE-SEM) analysis was used to conduct the morphological analyses of activated carbon. Fig. 2 displays the SEM images of AC before and after adsorption. The SEM image shows a high porous AC, which results in an increase in MB and Ph adsorption on the surface of AC. The rough surface of the prepared AC was visible in the micrographs (Fig. 2a–c) [47]. The images indicated an uneven leaf-like structure, a dispersed texture, and micropores on the surface of the activated carbon, which is due to pyrolysis-induced expulsion of volatile substances from the biomass. The presence of pores suggested possible adsorption sites [48]. The surface morphologies of the biomass waste activated carbon before adsorption with a catalytic agent solution ratio of 1:2 had a high number of distinct pores formed like honeycomb. The pores were produced as a result of the reaction between the carbon and the activating substance during the chemical activation process [49,50]. In addition, this result was correlated with the results of its high specific surface area, micropore volume, and total pore volume, as shown in Fig. 2. According to earlier research [51,52], the pore-creation process occurs, in general, due to the volatile molecules of the biomass waste and water molecules [53] during carbonization, creating an opening in the structure of the carbon. Fig. 2d and e shows the rough pores of AC after organic adsorption. The microgram shows the contaminant deposited on the activated carbon surface after adsorption (Fig. 2d and e) as compared to the initial samples (Fig. 2a, b, and 2c). These changes are due to the organic contaminant adsorbed onto the porous surface of the AC, leading to a rough porous surface [54]. The elemental composition by EDS analysis is also shown in Fig. 3. The EDS analysis showed high carbon content together with oxygen and hydrogen components, essential for high adsorption of phenol and methylene blue molecules onto the high porous material (Fig. 3a, b and 3c).

#### 3.1.3 BET surface area, pore volume and elemental analysis

**Fig. 4:**
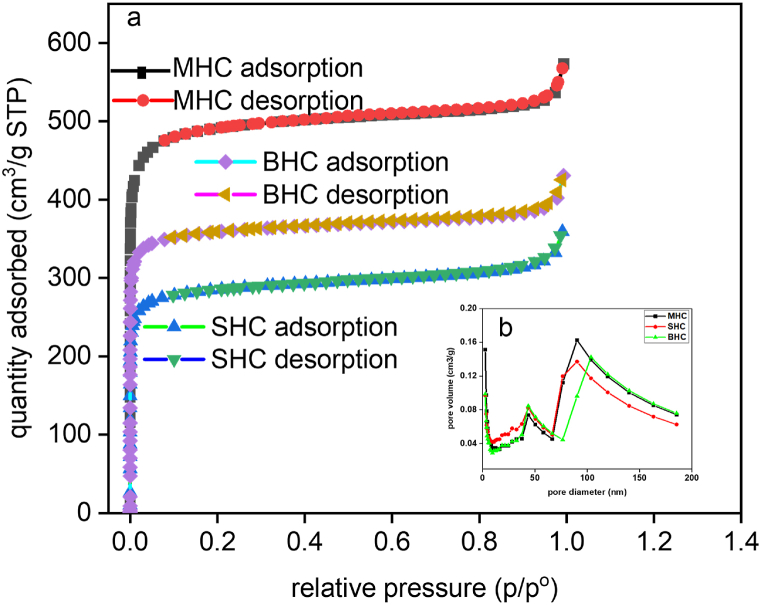
BET isotherm of MHC, BHC, and SHC.

The adsorption-desorption isotherms of the activated carbons are depicted in Fig. 4 to illustrate the pore configurations of the activated carbons and the related curves of pore size distribution. The samples showed a type I isotherm that exhibited materials with more micropores less than 2 nm as shown in Table 1. Similar results were observed by El Nemr et al. [55]. The adsorption-desorption isotherm demonstrated the coexistence of numerous micropores and significant mesopores in all three ACs with a quick and similar increase at P/P0 < 0.02, paired with noticeable hysteresis loops (P/P_o_ = 0.1–0.97). In addition, macropores were indicated by a small upward tendency at a high relative pressure (P/P_o_ = 0.97–1.0) [56]. Table 1 shows the surface area of the AC. The results showed the surface areas in the order of MHC > BHC > SHC. Determination of the elemental composition of AC was measured by CHN/O and TGA (Table 2). The results of CHN/O and TGA showed elemental carbon content in the order of MHC > BHC > SHC that confirmed to the EDS analysis (Fig. 3).

**Table 1:** BET isotherm of MHC, BHC, and SHC.

Adsorbent	BET surface area, m^2^/g	Mean pore diameter, nm	V_mic_, cm^3^/g	V_mes_, cm^3^/g	Total pore volume
MHC	1902.3	1.84	0.75	0.15	0.88
SHC	1115.9	1.99	0.41	0.14	0.56
BHC	1412.4	1.86	0.54	0.13	0.66

**Table 2:** CHNO analysis/TGA of activated carbon (MHC, BHC, and SHC).

Elemental analysis/Adsorbent	MHC	SHC	BHC
C(wt%)	73.39	54.79	69.70
H(wt%)	4.16	2.84	3.09
N(wt%)	0.71	0.76	0.62
O(wt%)	21.75	41.62	26.59
H/C	0.06	0.05	0.04
O/C	0.30	0.76	0.38
(O + N)/N	0.31	0.77	0.39
Fixed carbon	81.90	68.60	76.40
Volatile carbon	18.90	31.40	23.60

### 3.2 Adsorption study of phenol and MB

#### 3.2.1 Effects of initial concentration and effects of contact time

**Fig. 5:**
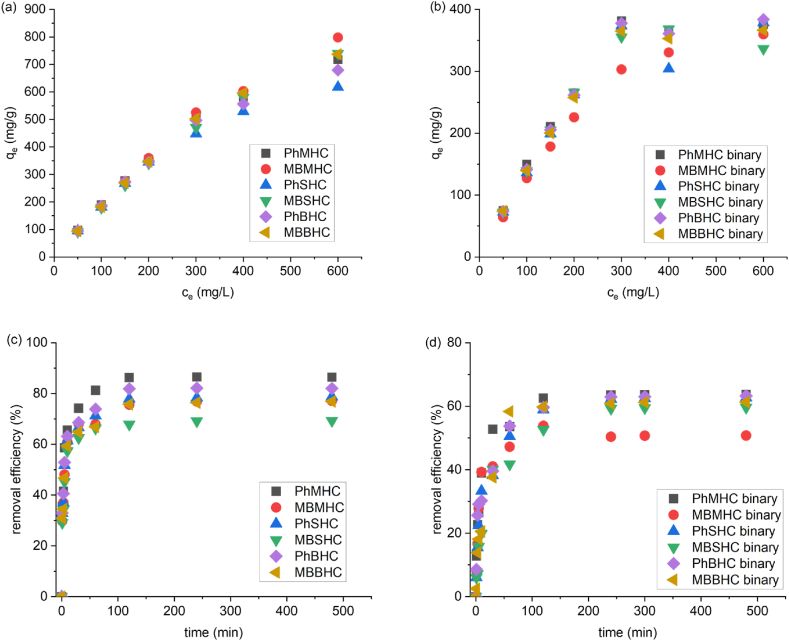
Effect of initial concentration, contact time onto MHC, SHC and BHC of mono (a and c) and binary adsorption systems (b and d).

The initial concentrations of phenol and MB were one factor influencing the adsorption efficiency and, subsequently, the removal efficiency. This investigation aimed to determine the impact of the initial phenol and MB concentrations on the MHC, BHC, and SHC adsorbents. This study was conducted at 30 °C with a fixed amount of adsorbent (0.5 g/L) for the single (Fig. 5a) and binary (Fig. 5b) adsorption. The adsorption capacity of phenol was higher than that of MB for both the single and binary adsorption.

At lower concentrations, the initial number of moles of phenol and MB to the accessible active surface area of the adsorbent was large for increased adsorption. As the concentration increased to a saturation point, at which the maximum organic solution uptake was observed and there were no open active sites left for more adsorption, there was an increase in the adsorption capacity for the adsorbent dosage used. Similar findings were reported in the literature [57,58]. When the initial phenol and MB concentrations increased at adsorbents’ equilibrium, then the MB adsorption capacity (q_t_) increased [59]. The maximum adsorption capacity of the initial concentration of 50–600 mg/L MB was 92.5–798.0 mg/g in the single system and 64.2–359.8 mg/g in the binary system on the MHC, BHC, and SHC adsorbents [60]. The maximum adsorption capacity of phenol at initial concentrations of 50–600 mg/L was 95.1–717.2 mg/g in the single system and 72.7–383.8 mg/g in the binary system. However, this result could be explained by the fact that a greater amount of aqueous MB and phenol interacted with the active sites of the adsorbent, and the phenol and MB concentration increased, which in turn caused the adsorption process to proceed more rapidly [61]. Hence, the adsorption mechanism could be influenced by the initial phenol and MB concentrations in the aqueous solution [62].

One of the most important variables in transfer processes, including adsorption, is contact time. Therefore, it is important to investigate the optimum contact time, which affects the adsorbents’ ability to retain contaminants. The adsorption capabilities for phenol and MB were measured as a function of time (single and binary systems) to determine the acceptable contact time for phenol and MB adsorption, and the influence of contact time on the phenol and MB removal efficiency of single and binary systems are shown in Fig. 5c and d. The influence of contact time on adsorption efficiency was investigated at 30 °C, 0.5 g/L, and 1–480 min as shown in Fig. 5c and d. The MB and phenol uptake increased rapidly during the first 30 min before gradually increasing until saturation, and equilibrium adsorption was established after 120 min, resulting in a removal efficiency greater than 80 % in the single system and more than 50 % in the binary system. This may have been the result of the unoccupied active sites of the absorbent surface area being available for adsorption initially and gradually being reduced with an increase in time. After that time, there were not many surface-active sites available, therefore, there was not much of an increase in the phenol and MB uptake. The presence of hydroxyl groups (Fig. 1) on the activated carbon surface also enhanced the initial rapid adsorption due to electrostatic attraction and the creation of hydrogen bonds [63]. Similar findings were observed by Tran et al. [64]. The time needed to establish equilibrium was chosen as the contact time for further trials, as illustrated in Fig. 5. Furthermore, the rapid phenol and MB uptake at 120 min for the binary adsorption confirmed the large specific surface areas, pore volumes, and porous structures on the activated carbon in this study as shown in Table 1. This result agreed with the BET analysis showing surface areas in the order of MHC > BHC > SHC and adsorption efficiencies in the order of MH > BHC > SHC.

#### 3.2.2 Effects of adsorbent dosage

**Fig. 6:**
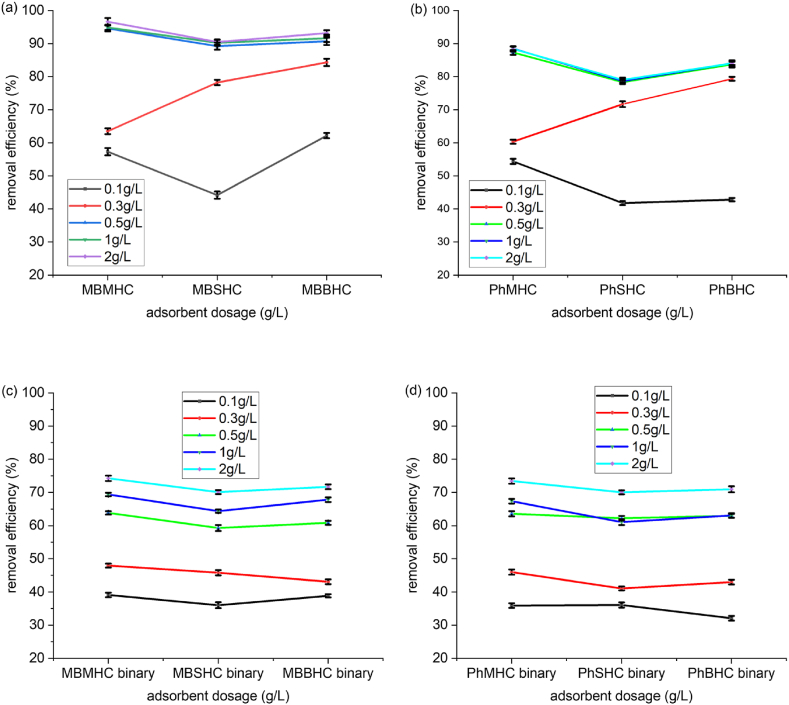
Effect of adsorbent dosage of MHC, SHC and BHC for (a) MB, (b) Phenol, (c) MB binary and (d) Phenol binary adsorption.

The adsorbent dosage had a significant impact on adsorption efficiency. The capacity of the adsorbent for phenol and MB was determined by the distribution between the adsorbent and the aqueous solution when the system was in an equilibrium state. As the amount of the adsorbent material increased, the uptake increased. Adsorption increased significantly when the carbon dosage increased from 0.1 to 2 g/L of 300 mg/L contaminant for both single and binary adsorption (Fig. 6a, b, 6c, and 6d). These results showed that the removal efficiency of phenol and MB for the single adsorption was >70 % and it was <65 % for the binary adsorption. The removal efficiency between the adsorbents also confirmed that MHC had a higher potential for both phenol and MB adsorption. After 0.5 g/L of adsorbent, there was no significant increase in the adsorbate removal (Fig. 6). From this perspective, the amount of AC was held constant at 0.5 g/L in all of the following experiments [65]. The less significant increase in removal efficiency of adsorbent after 0.5 g/L could have resulted for two reasons: (i) As a consequence of the repulsive forces between molecules of adsorbate on the adsorbent surface and in the bulk phase, further absorption on the remaining unoccupied surface sites was prevented. As a result, the primary drive for mass exchange between the aqueous solution (phenol and MB adsorbate) and the adsorbent dosage did not increase significantly with time. (ii) The phenol and MB must have covered greater distances and penetrated deeper into the pores while encountering much greater resistance [66]. The competition of both phenol and MB in the binary system contributed to the decrease in removal efficiency compared to that in the single system. Similar results from Mubarik et al. [67] indicated a removal percentage of 95 % of phenol (concentration of 25 mg/L solution) from 1 g/L of sheesham sawdust activated carbon and Jawad et al. [68] also obtained a removal efficiency of 83 % MB (concentration of 0.1 g/100 mL) from 1 g/L of coconut shell AC.

#### 3.2.3 Determination of pH_PZC_ and effect of pH

**Fig. 7:**
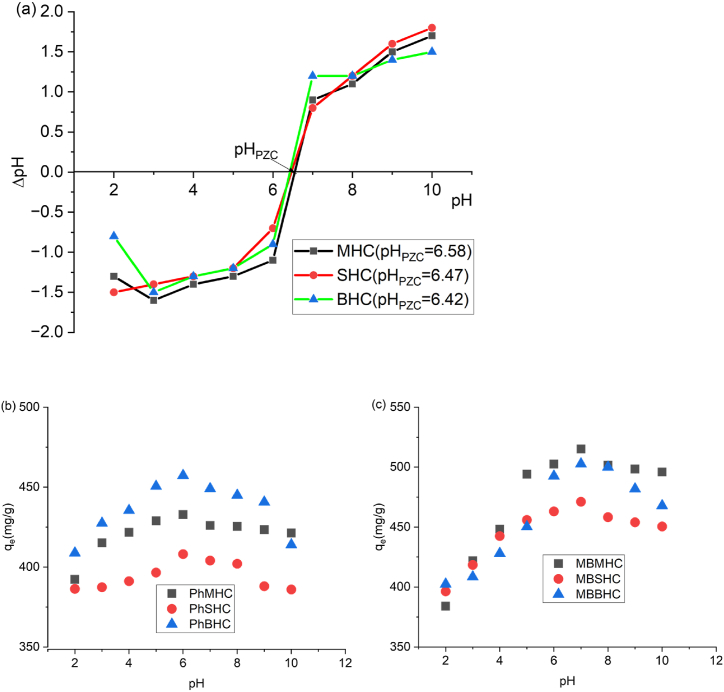
(a) pH_PZC_ onto activated carbon (MHC, SHC and BHC)*,* (b) effect of on Phenol adsorption and (c) effect of pH on MB adsorption.

The measurement for pH was determined from the AC's surface exhibiting a zero charge is called pHpzc. A positive net charge of AC occurs when the pH < PZC, whereas a negative net charge occurs when the pH > PZC. In Fig. 7a the prepared ACs in this study had a zero-point charge (pH_ZPC_) of MHC (6.58), SHC (6.47) and BHC (6.42). This indicates that the surface charge of AC is positive for pH values less than 6.42 and negative for pH values greater than 6.58 [69,70].

The effect of pH (2-10) on the adsorption of phenol and basic dye (methylene blue) from aqueous solution onto MHC, SHC and BHC is depicted in Fig. 7b and c. It was shown that the pH of the solution had a significant impact on the adsorption process, influencing both the extent of ionization of the adsorbate and the surface charge of the adsorbent. As a result of decreased electrostatic attract ions between positively charged MB dye anions and positively charged adsorption sites and ionic repulsion between the positively charged surface and the cationic dye molecules [71]. There will be high MB adsorption at pH greater than 6.42 (pH_ZPC_) as indicated with the optimum pH of 7 (Fig. 7a). Similar results were observed by Ebrahimian Pirbazari et al. [72]. pH values less than 6.58 (pH_ZPC_) depicts an optimum pH of 6. The presence of extra H^+^ ions competing with the cation groups on the MB dye for adsorption sites is likely the cause of the lower MB adsorption at acidic pH [73]. The negatively charged surface of AC particles may increase the positively charged of MB dye cations by electrostatic forces of attraction at higher pH values. The opposite was observed with phenol adsorption as the H^+^ released at lower pH promote increased adsorption [74].

### 3.3 Proposed mechanism of activated carbon production using KOH

Fig. S1 displays the possible chemical activation mechanism with KOH during activation and the gaseous products released during activation such as CO, CH_4_, H_2_, H_2_O vapor and CO_2_. After reaction of biomass waste (MH, SH or BH) with KOH, H_2_O is released under heat which aids in producing char and tar. Char reacts with vapor H_2_O under heat to release H_2_, CO and CO_2_ [75]. The K_2_O and the biomass waste at higher temperatures further released gases to produce porous carbon (C) [76,77].

### 3.4 Adsorption kinetics

**Fig. 8:**
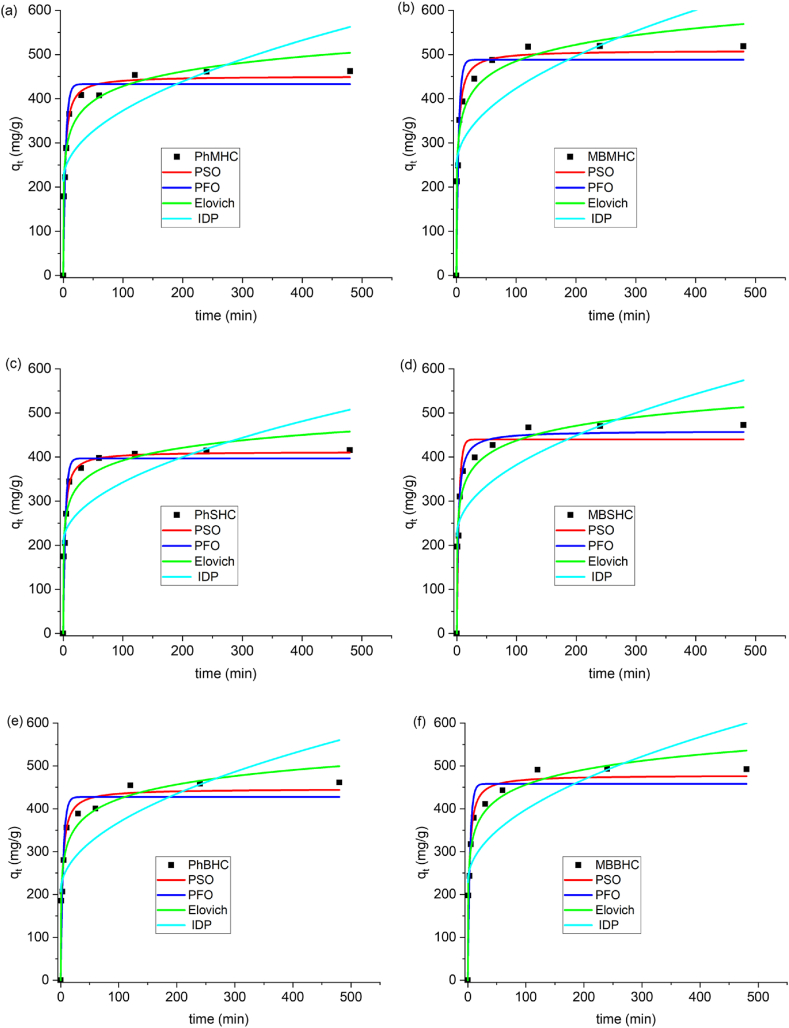
Mono-adsorption kinetic model for phenol and MB onto MHC, SHC and BHC.

The underlying mechanism, which is equivalent to the slowest reaction step, can provide more knowledge than may be acquired from this study four alternative experimental data were fitted to the non-linear kinetic models in the study of the kinetic sorption data: the pseudo-first order (Eq. (3)), pseudo-second order (Eq. (4)), Elovich (Eq. (5)), and intra-particle diffusion (Eq. (6)) models. Fig. 8 each displays the parameters and results of non-linear fitting of the adsorption kinetic models. For kinetic analysis, the experimental data from the single adsorption experiments were employed as shown on Table 3.

**Table 3:** Kinetic models for phenol and MB adsorption parameters.

Adsorbent	PFO			PSO		
	K_1_ (1/min)	q_e_ (mg/g)	R^2^	K_2_ (g/mg/min)	q_e_ (mg/g)	R^2^
PhMHC	0.25	432.96	0.9438	0.00089	451.24	0.9785
PhSHC	0.26	397.00	0.9462	0.00100	412.15	0.9791
PhBHC	0.23	427.47	0.9245	0.00085	446.28	0.9652
MBMHC	0.26	488.30	0.9300	0.00084	509.47	0.9741
MBSHC	0.26	440.24	0.9265	0.00094	458.90	0.9693
MBBHC	0.26	458.11	0.9278	0.00089	478.36	0.9732

	**Elovich**			**IDP**		
	A	B	R^2^	c	k	R^2^
PhMHC	3336.38	0.020	0.9614	213.95	15.90	0.5622
PhSHC	4965.40	0.023	0.9504	204.35	13.82	0.5183
PhBHC	2767.45	0.020	0.9659	206.93	16.12	0.5876
MBMHC	4605.46	0.018	0.9638	246.14	17.71	0.5531
MBSHC	4229.32	0.020	0.9655	221.40	16.08	0.5623
MBBHC	3977.78	0.019	0.9712	228.93	16.92	0.5716

The results showed that (Fig. 8), for all initial phenol and MB concentrations, the pseudo-second-order kinetic model (Fig. 8a, b, 8c, 8d, 8e and 8f) had the highest correlation coefficients (R^2^) of 0.9652–0.9791 and 0.9693–0.9741, respectively. Therefore, the pseudo-second-order model best described the kinetic data from the non-linear single adsorption showing that the adsorbents of phenol and MB adsorption were mostly chemisorption. The adsorption capabilities predicted by the pseudo-second order kinetic model were also substantially closer to the experiment's values [78]. Due to the good fit (Fig. 8a, b, 8c, 8d, 8e and 8f) of the PS0 and Elovich equation, adsorption depicts chemisorption process of MB and phenol onto MHC, SHC and BHC [79,80]. The intra-particle diffusion model was then applied to the experimental data. This helped to further understand the mechanisms involved in pollutant transfer from the adsorbate phase to the surfaces of MHC, SHC, and BHC. A high value for intra-particle diffusion was observed, which may have been advantageous for the adsorption process. This is due to the easy release of adsorbate molecules from the bulk phase into the solution, leading to increased adsorption. A greater c value (Eq. (6)), on the other hand, may indicate increased adsorption resistance. Therefore, resulting in the decreased in the intra-particle diffusion model correlation coefficients <60 % [42].

(3) $$q_{t}=q_{e\;}\left(1-e^{-k_{1}t}\right)$$

(4) qt=ktqe2t1+k2qet

(5) $$q_{t}=1/B\left(\ln\left(ABt+1\right)\right)$$

(6) $$q_{t}=kt^{\frac{1}{2}}+c$$

where q_e_ represents the amounts of adsorbate adsorbed at time t and equilibrium (mg/g), respectively; k_1_ and k_2_ correspond to the PFO and PSO rate coefficients, respectively (Fig. 8a, b, 8c, 8d, 8e and 8f). The pseudo-second-order kinetic model (Fig. S2) best fitted the binary experimental data indicating a chemisorption reaction onto MHC, BHC and SHC. The adsorption data of the phenol in the presence of methylene blue was also determined by Mubarik et al. [67] to be the best fit for the pseudo-second-order kinetic model (Table S1). The q_t_ represents the amounts of adsorbate adsorbed at time t, A refers to initial adsorption rate (mg/g min), and B (mg/g min), refers to desorption constant for each experiment. Adsorption rate increases exponentially as the adsorbed molecules decrease on the surface of AC. As the amount of adsorbed molecules increases, the biosorption rate decreases exponentially as per Elovich model. From Table 3 and Table S1, R^2^ values are high and closer to R^2^ value of PSO indicating chemisorption adsorption. K (Eq. (6)) is the intraparticle diffusion (IPD) rate constant (mg/g min^1/2^). Low values of IPD R^2^ (0.5183–0.5876) were observed (Table 3 and Table S1) implying that the adsorption model of IPD did not suite the experimental adsorption data.

#### 3.4.1 Adsorption isotherm for mono adsorption and Ph-MB binary adsorption system

**Fig. 9:**
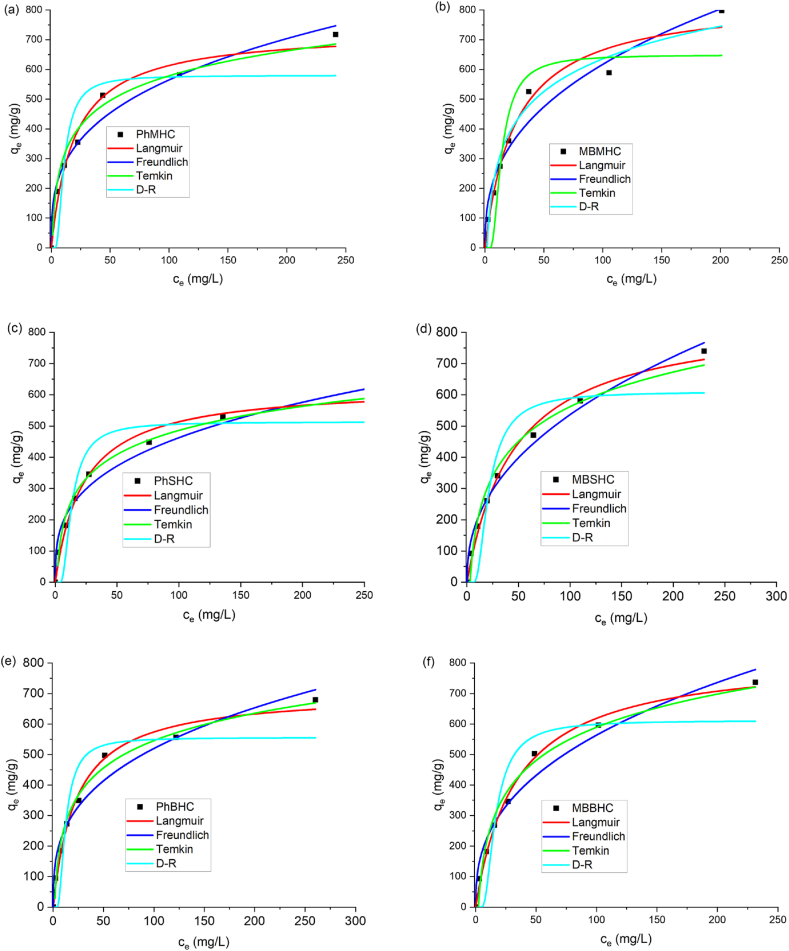
Mono-adsorption isotherm model for Phenol and MB onto MHC, SHC and BHC.

Fig. 9 and Fig. S3 depict the phenol and MB adsorption isotherms on AC for the mono-adsorption and binary adsorption systems. The adsorption capacities were obtained using the adsorption isotherms: Langmuir (Eq. (7)), Freundlich (Eq. (8)), Temkin (Eq. (9)), and Dubinin–Radushkevich (Eq. (10)).

The Langmuir adsorption isotherm has been widely employed for the sorption of a solute from a liquid solution and has been effectively used in several pollutant adsorption procedures. The Langmuir isotherm in its most typical form is shown in Eq. (7).

(7) qe=qmkLce1+KLce

where q_m_ denotes the maximum adsorption capacity (mg/g), q_e_ is the equilibrium capacity for a complete monolayer (mg/g), k_L_ is a constant linked to the adsorption capacity, K_L_ is a constant related to the binding site affinity and adsorption energy (L/mg), and c_e_ is the equilibrium concentration (mg/L). The adsorption onto a heterogeneous surface is described by the Freundlich isotherm, which is an empirical equation. The Freundlich isotherm is frequently expressed as shown in Eq. (8) as follows

(8) qe=kfCe1n

where K_f_ (mg/g)(L/mg)^1/n^) and 1/n are the Freundlich constants relating to the adsorbent's adsorption capacity and intensity, respectively. The Temkin isotherm computes the factors that are directly related to the interactions of the adsorbents and adsorbates. Temkin model is specified by the equally distributed binding energy (up to a specific maximum binding energy), which is derived by graphing the adsorption capacity at equilibrium (q_e_) versus lnC_e_. The non-linear equation for Temkin isotherm adsorption is given by Eq. (9).

(9) $$q_{e}=B\;\ln\left({Ac}_{e}\right)$$

where,B=RTb

T is the absolute temperature, R is the gas constant (8.314 J/mol K), and B is the heat adsorption constant, with the value defined by the gradient and intersection with the y-axis. While b depicts Temkin constant (KJ/Kgmol)(g/mg) and A is Temkin equilibrium binding energy constant. The Temkin isotherm constant (L/mg) is determined by the maximal binding energy of adsorbate and adsorbate.

Adsorption is assumed to have a multilayer structure in the Dubinin-Radushkevich (D-R) isotherm model, which includes Van der Waals forces that indicate physical adsorption processes. The Dubinin-Radushkevich (D-R) isotherm assumed to have a multilayer structure and applied to assess the adsorption process of van der Waals forces to verify if it was physisorption or chemisorption. The non-linear model of the Dubinin-Radushkevich equation is as shown in Eq. (10):

(10) qe=qmexp−β(RTIn(1+(1ce)))2

(11) ε=RTIn(1+1ce)

Where ℇ is the Polanyi potential (kJmol^−^^1^) (Eq. (11)), q_m_ (mg/g) is the adsorption capacity, β is the D-R constant, T(K) is the absolute temperature, R is the gas constant 8.31Jmol^−1^k^−1^.

(12) E=1√2β

Equation (12) states that physical adsorption occurs if the energy (E) value estimated from the D-R isotherm is < 8 kJ/mol. Adsorption occurs chemically if this value is between 8 and 16 kJ/mol [81]. The energy value in this study was <8 JKmol^−1^ indicating the reaction is physical at AC and the contaminant surface adsorption. Fig. 9a, b, 9c, 9d, 9e, and 9f present the graphs of experimental data and Table 4 shows the corresponding parameters for the fitted models. The evaluation of each isotherm was assessed using the R^2^ values of Langmuir (0.9769–0.9953), Freundlich (0.9657–0.9898), Temkin (0.9729–0.980), and D-R (0.8600–0.8753) for MB adsorption, as well as for phenol, R^2^ of Langmuir (0.9790–0.9901), Freundlich (0.9677–0.9795), Temkin (0.9776–0.9908), and D-R (0.8324–0.8872) adsorption. Based on the correlation coefficient, the Langmuir model best matched the experimental data for the adsorption of MB onto MHC, SHC, and BHC. The Langmuir model with monolayer adsorption was observed, implying that there was contact between the surface of the adsorbent and the adsorbate molecules. This phenomena was also observed by Zhang et al. [82]. This indicates that there is uniform binding energy for the MB and phenol adsorbed on the microporous/mesoporous surface of the AC. The homogeneous distribution of active sites on the AC adsorbent surface constitutes physical adsorption throughout the surfaces of the AC and monolayer coverage (Table 4). In addition, K_L_ values were in 0.02–0.05 range which is within the favorable limit, hence adsorption hypothesis above was supported. The high maximum adsorption capacity of the MB and phenol shows that the activated carbon produced with KOH produced high surface area, surface functional groups and surface morphology for monolayer adsorption. The n value between 2 and 4 in the Freundlich depicts a favorable condition for adsorption on AC though the correlation coefficient of Temkin > Freundlich > D-R isotherms. Furthermore, the maximum adsorption capacity (q_m_) values obtained for the activated carbon as adsorbent when compared with present research in the literature, they will significantly add to the literature. However, the current studies were conducted to determine whether the readily accessible, low-cost adsorbents activated under same condition and KOH as activated agent is suitable in adsorption or be used to treat MB and phenol efficiently.

**Table 4:** Isotherm models for phenol and MB adsorption parameters.

Adsorbent	Langmuir			Freundlich		
	K_L_ (L/mg)	q_m_	R^2^	K_f_ (mg/g)(L/mg)^1/n^	n	R^2^
PhMHC	0.05	731.56	0.9790	131.52	3.15	0.9775
PhSHC	0.04	629.96	0.9869	108.09	3.16	0.9795
PhBHC	0.04	705.09	0.9901	114.20	3.03	0.9677
MBMHC	0.03	838.42	0.9769	107.07	2.62	0.9657
MBSHC	0.02	855.96	0.9924	74.46	3.15	0.9898
MBBHC	0.02	826.28	0.9953	95.60	2.59	0.9714

	**Temkin**			**D-R**			
	A	B	R^2^	q_m_	E	k	R^2^
PhMHC	0.21	120.53	0.9776	580.06	2.30	0.09	0.8324
PhSHC	0.78	111.28	0.9908	513.22	1.87	0.14	0.8872
PhBHC	0.68	129.20	0.9897	555.95	2.03	0.12	0.8741
MBMHC	0.55	158.29	0.9729	649.37	1.78	0.15	0.8741
MBSHC	0.33	160.18	0.9813	609.92	1.19	0.35	0.8600
MBBHC	0.42	156.82	0.9859	611.60	1.52	0.21	0.8753

Experiments on the adsorption of phenol and methylene blue were derived as the effects on phenol or MB adsorption with solution (1:1) containing phenol-methylene blue (Ph-MB) coexisting (binary-adsorption) in the solution (Fig. S3). When phenol and MB coexisted in a system, the removal efficiency by the activated carbon decreased compared to that in the single adsorption system. Due to competitive sites for both organic contaminants (Ph and MB) on the activated carbon, the removal efficiency decreased. The maximum adsorption capacity also significantly decreased due to competitive adsorption and adsorbent active sites of both the phenol and methylene blue onto the adsorbents. In the binary-adsorption system, the Freundlich isotherm fitted (Table S2) best having a correlation value 0.9775–0.9931 > 0.9587–0.9912 (Temkin) > 0.9424–0.9895 (Langmuir) > 0.7956–0.8465 (D-R) isotherms (Fig. S3).

#### 3.4.2 Effect of temperature and thermodynamic interpretation

**Fig. 10:**
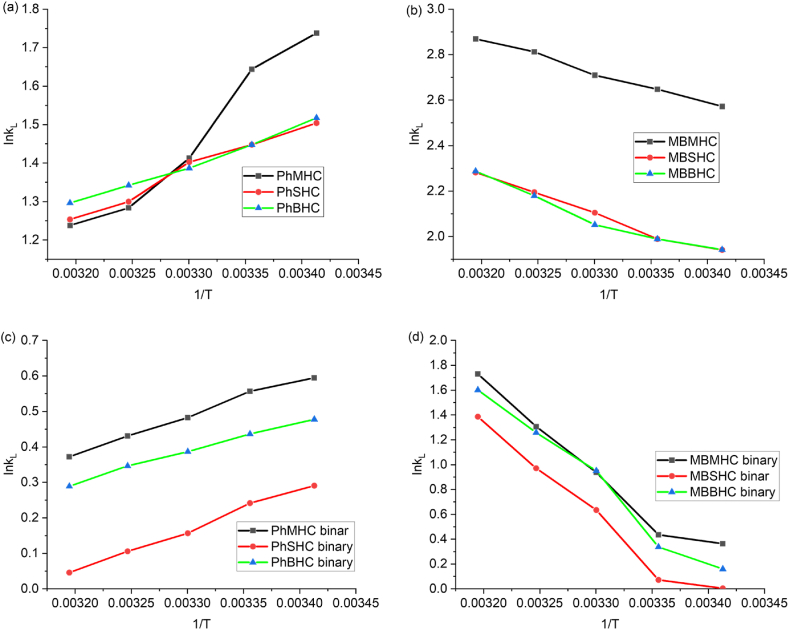
Temperature effect of mono and binary-adsorption of phenol and methylene blue.

For the adsorption of organic contaminants, temperature is another crucial factor. It is preferable to get a high equilibrium adsorption at room temperature without using more energy. A temperature range of 20–40 °C was used to study temperature effect on the equilibrium adsorption. At pH 7 (MB) and pH 6 (Phenol), the experiments were conducted of 0.5 mg AC and an initial MB and Ph concentration of 300 mg/L. When the temperature was raised from 20 to 40 °C, AC adsorption capacity of MB increase and phenol decrease (Fig. 10a and b) for both mono and binary adsorption systems. Similar results were observed for the binary system (Fig. 10c and d). This indicated that MB uptake was preferred at higher temperatures and phenol adsorption preferred at lower temperatures. Phenol adsorption decrease seen with increasing temperature could perhaps be attributed to the attenuation of adsorptive forces between the adsorbent and adsorbate active sites of species [83].

The observed increase in adsorption of MB with increase in temperature could be as follows: (i) at high temperature, dye mobility increased to penetrate AC pores; (ii) increased chemical interactions between the activated carbon's surface functionalities and the adsorbate; or (iii) a chemical potentials shift associated with the adsorbate species' solubility [84]. As a result, the solubility and temperature effects both have the same overall effect, hence, the nature of the adsorption process is endothermic [83,85].

In the investigation of adsorption thermodynamics, the temperature influence and adsorption mechanism were studied. An analysis was conducted on the thermodynamics of MB and phenol adsorption on AC from an energy perspective. The adsorption process was found to be spontaneous or not by investigating the driving force of adsorption using the adsorption thermodynamics approach. Equations (13), (14), (15) and 16 provide the thermodynamic formulae that are typically used to compute the values of thermodynamic parameters, such as free energy change (ΔG), enthalpy change (ΔH), and entropy change (Δ*S*) [86].

(13) $$\Delta G=-RTlnk_{L}$$

(14) $$k_{L}=\frac{q_{e}}{c_{e}}$$

(15) $$\ln\;k_{L}\frac{\Delta S}{R}-\frac{\Delta H}{RT}$$

(16) ΔG=ΔH−TΔS

where k_L_ is the thermodynamic constant, T is the absolute temperature (K), and R is the universal gas constant (8.314 Jmol^−1^K^−1^). The Gibbs free energy change ΔG (kJmol^−1^), the entropy changes ΔS (kJmol^−1^K^−1^), and the enthalpy change ΔH (kJ·mol^−1^). The determination of the intercept and the slope from linear graph of lnk_L_ vs 1/T, as shown in Fig. S4 were used to calculate the values of ΔS and ΔH respectively and the values of ΔG, ΔH and ΔS, reported in Table 5. An endothermic reaction is suggested by the positive value of ΔH and exothermic by negative value of ΔH [63]. The adsorption of organic contaminant onto AC is suggested to be more random disorder at the solid/solution interface when ΔS is positive and less random when ΔS is negative. The adsorption process appears to be spontaneous based on the negative values of ΔG. Moreover, the decrease in ΔG values as temperature rises suggests that adsorption is more spontaneous at higher temperatures for MB than Phenol. The increased in ΔG values of phenol as temperature decreases indicates that the process is less spontaneous at higher temperature. The same trends were observed for the binary adsorption of MB and Ph (Table 6). For physisorption, the change in free energy is typically between (−20 and 0) kJmol^−1^, whereas chemisorption occurs in the range of (−80 to 400) kJ mol^−1^. The ΔG values for mono and binary adsorption of MB and Ph were between −8 kJ/mol and 0 kJ/mol showing the process was controlled by physical and spontaneous adsorption [87].

**Table 5:** Thermodynamic parameters of mono-adsorption of Ph and MB.

Adsorbent	T(K)	Δ G(KJmol^−1^)	ΔH (KJmol^−1^)	ΔS (Jmol^−1^k^−1^	R^2^
PhMHC	293	−4.23	−20.79	−56.49	0.9595
	298	−4.07			
	303	−3.55			
	308	−3.28			
	313	−3.22			
MBMHC	293	−6.26	11.55	60.78	0.9925
	298	−6.56			
	303	−6.82			
	308	−7.20			
	313	−7.46			
PhSHC	293	−3.66	−9.87	−21.13	0.9792
	298	−3.58			
	303	−3.53			
	308	−3.32			
	313	−3.26			
MBSHC	293	−4.72	13.51	62.09	0.9864
	298	−4.92			
	303	−5.30			
	308	−5.62			
	313	−5.93			
PhBHC	293	−3.69	−8.36	−15.98	0.9931
	298	−4.01			
	303	−4.33			
	308	−4.67			
	313	−4.99			
	293	−3.69			
MBBHC	293	−4.73	13.39	61.59	0.9574
	298	−4.92			
	303	−5.16			
	308	−5.58			
	313	−5.95

**Table 6:** Thermodynamic parameters of binary-adsorption of Ph and MB.

Adsorbent	T(K)	Δ G(KJmol^−1^)	ΔH (KJmol^−1^)	ΔS (Jmol^−1^k^−1^	R^2^
PhMHC	293	−1.44	−8.69	−24.66	0.9915
	298	−1.37			
	303	−1.21			
	308	−1.10			
	313	−0.96			
MBMHC	293	−0.88	54.79	188.89	0.9606
	298	−1.07			
	303	−2.36			
	308	−3.34			
	313	−4.50			
PhSHC	293	−0.70	−9.53	−30.09	0.9935
	298	−0.59			
	303	−0.39			
	308	−0.27			
	313	−0.11			
MBSHC	293	−0.01	55.724	189.11	0.9608
	298	−0.17			
	303	−1.59			
	308	−2.48			
	313	−3.60			
PhBHC	293	−1.16	−7.12	−20.30	0.9945
	298	−1.08			
	303	−0.97			
	308	−0.88			
	313	−0.75			
MBBHC	293	−0.38	57.97	198.60	0.9771
	298	−0.83			
	303	−2.39			
	308	−3.22			
	313	−4.16			

The adsorption process ΔH for mono and binary adsorption of MB were positive, indicating an endothermic reaction, whereas negative ΔH for mono and binary adsorption for Ph were exothermic reaction (Table 5, Table 6). Negative ΔS values of mono and binary adsorption of MB were negative suggesting there is decreased randomness during adsorption at the liquid-solid interface. The positive ΔS for mono and binary adsorption of Ph indicated increase in randomness and disorder at the liquid -solid interface [88].

### 3.5 Propose adsorption mechanism of phenol and MB mechanism onto activated carbon

**Scheme 1:**
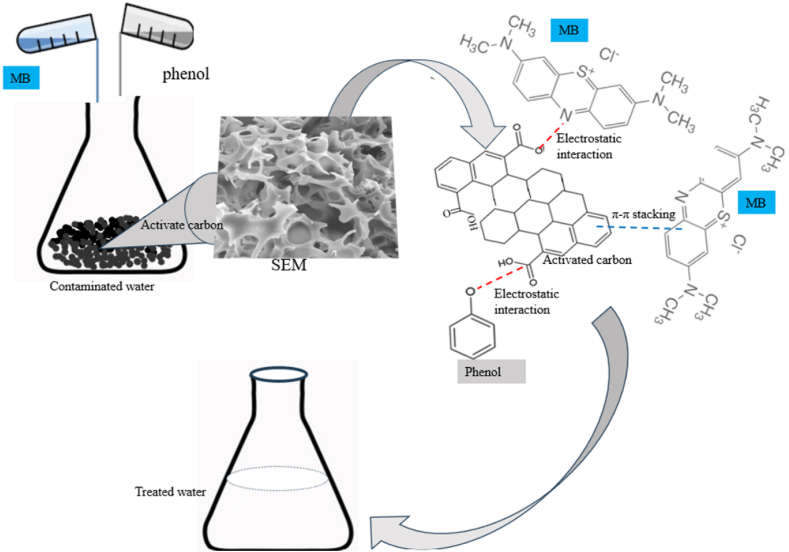
Proposed adsorption mechanism for Phenol and MB on activated carbon.

Surface area and surface chemistry properties of the adsorbent surface (AC) significantly influence the adsorption of contaminants [89,90]. The surface morphology analysis of MHC, SHC, and BHC has a large surface area, supported by well-developed micropores (Fig. 2, Fig. 4). A network of micropores dominated the AC surface and the mean pore diameter is between 1.86 nm and 1.99 nm (Table 1) which allowed methylene dye and phenol molecules with a molecular size of 0.84 nm and 0.75 nm, respectively to diffuse into the internal pores of the AC [[91], [92], [93]]. Mechanisms for phenol and MB adsorption include electron donor-acceptor interaction, hydrogen bonding, π-π attraction, and electrostatic attraction in the presence of appropriate functional groups (-COO-, -O, -OH) on the AC surface (Scheme 1) [[94], [95], [96]].

### 3.6 Regeneration and reusability

**Fig. 11:**
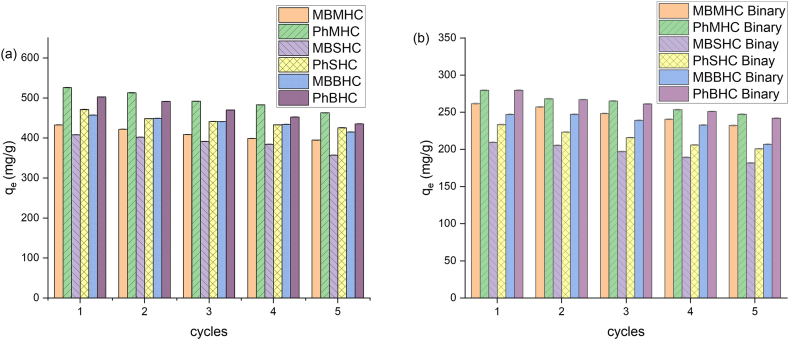
Regeneration of spent adsorbent under the adsorption conditions of phenol and MB concentration 300 mg/L and temperature of 25 °C.

Used/spent adsorbents, often waste, can cause environmental contamination due to toxic compounds. Alternatives include desorption and regeneration of spent adsorbents, which promote environmentally friendly practices by reducing the need for disposal and minimizing hazardous waste. Chemical regeneration was used in this study which refers to an in-situ method that uses chemical reagents that facilitate adsorbate desorption from activated carbon. Chemical regeneration is a method that effectively removes contaminants by recovering activated carbon saturated with contaminants, offering benefits such as targeted removal, cost-efficiency, and efficacy. The growing economic and financial need for sustainability makes regeneration studies crucial for assessing the potential of adsorbent reusability in practical use. Five cycles of the adsorption–desorption procedure was used to study the regeneration of MHC, SHC, and BHC; the adsorption capacity in each cycle is shown in Fig. 11 for the mono and binary regeneration system. Fig. 11a shows the desorption study with five cycles using a 0.01M HCl, rinsed in warm distilled water and dried at 105 °C [97,98]. It was observed that MHC, SHC and BHC adsorption capacity decreases with each subsequent cycle. MHC, SHC and BHC demonstrated a starting Ph adsorption capability of 525.58 mg/g, 471.08 mg/g, and 502.66 mg/g, respectively and decreased to 462.88 mg/g, 425.54 mg/g, and 435.54 mg/g after five cycles (Fig. 11a). MHC, SHC and BHC demonstrated a starting Ph binary adsorption capability of 279.58 mg/g, 233.38 mg/g, and 279.58 mg/g, respectively and decreased to 247.22 mg/g, 200.94 mg/g, and 242.10 mg/g after five cycles (Fig. 11b). Similar results were observed for MB mono and binary adsorption-desorption graphs. The findings of the desorption demonstrated that MHC, SHC and BHC could be reused and regenerated in wastewater treatment relating to Kamdod and Kumar [39] studies.

## 4 Conclusions

Activated carbon was produced from *M. oleifera*, baobab, and sesame husks (MHC, BHC, and SHC, respectively) by chemical activation for MB and phenol adsorption. Hydroxyl groups were observed on the surface of the activated carbon in the FTIR results, while the results of SEM analysis agreed with BET analysis and showed significant micro and meso-pores on the surface morphology on SEM analysis. The MHC, SHC, and BHC with high surface areas fitted best with the Langmuir isotherm and pseudo-second order reaction and, therefore, a monolayer chemisorption adsorption for the single system. Adsorption in binary Ph-MB system was confirmed as multilayer Freundlich chemisorption, with lower adsorbate adsorption compared to single system due to competitive sites of both organics. Regeneration studies showed that the AC materials are potential adsorbent for reusability.

## CRediT authorship contribution statement

**Numfor Linda Bih:** Writing – review & editing, Writing – original draft, Validation, Methodology, Investigation, Conceptualization. **Mwemezi J. Rwiza:** Software, Conceptualization. **Asha S. Ripanda:** Validation, Resources, Methodology, Conceptualization. **Assia Aboubakar Mahamat:** Validation, Conceptualization. **Revocatus L. Machunda:** Supervision, Methodology, Investigation. **Joon Weon Choi:** Validation, Supervision, Methodology, Funding acquisition.

## Data availability statement

Data will be made available on request.

## Declaration of competing interest

The authors declare that they have no known competing financial interests or personal relationships that could have appeared to influence the work reported in this paper.
