# Supplementary material for: Adsorption of phenol and methylene blue contaminants onto high-performance catalytic activated carbon from biomass residues
Source: Heliyon. 2024 Dec 12;11(1):e41150. doi: 10.1016/j.heliyon.2024.e41150 (PMC11721239; doi:10.1016/j.heliyon.2024.e41150)
Supplement: Multimedia component 1 [file mmc1.docx]

**Supplementary data**

**Adsorption of phenol and methylene blue contaminants onto high-performance catalytic activated carbon from biomass residues**

Numfor Linda Bih^a,b^*, Mwemezi J. Rwiza^a^, Asha S. Ripanda^a^, Assia Aboubakar Mahamat^c^, Revocatus L. Machunda^a^, Joon Weon Choi^b^*

^a^School of Materials, Energy, Water and Environmental Sciences (MEWES), The Nelson Mandela African Institution of Science and Technology (NM-AIST), School of Materials, Energy, Water and Environmental Sciences (MEWES), P. O. Box, 447, Arusha, Tanzania

^b^Graduate School of International Agricultural Technology, Department of Green Eco System

Engineering, Seoul National University, Pyeongchang 25354 Gangwon-do, South Korea

^c^Nile University of Nigeria: Abuja, Federal Capital Territory, Airport Rd, Jabi 900001, Abuja, Nigeria

*Corresponding author: E-mail: numforl@nm-aist.ac.tz

cjw@snu.ac.kr

**Figures caption**

**Fig. S1:** Proposed adsorption mechanism for catalytic activation of biomass residues AC

**Fig. S2:** Kinetic model for binary-adsorption (Ph-MB) onto MHC, SHC and BHC

**Fig. S3:** Isotherm models of Ph-MB binary-adsorption system

**Fig. S4:** Thermodynamic models of Ph-MB binary system


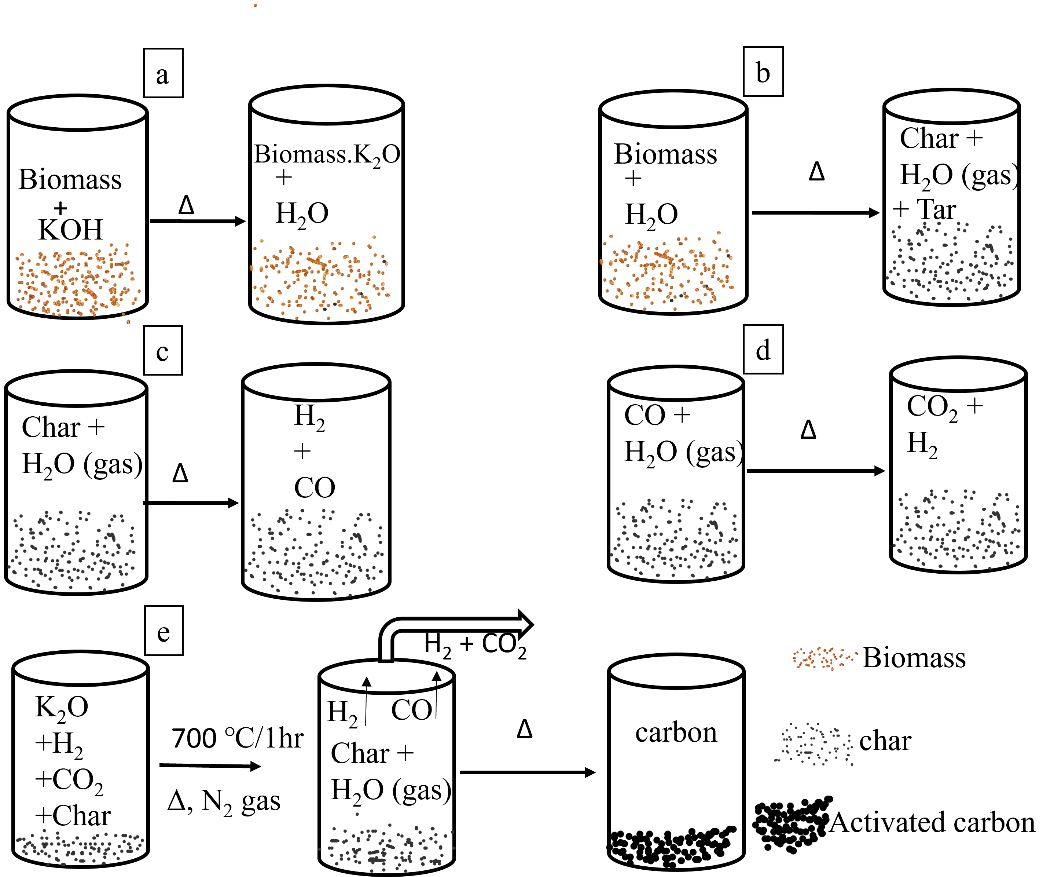


**Fig. S1:** Proposed adsorption mechanism for catalytic activation of biomass residues AC


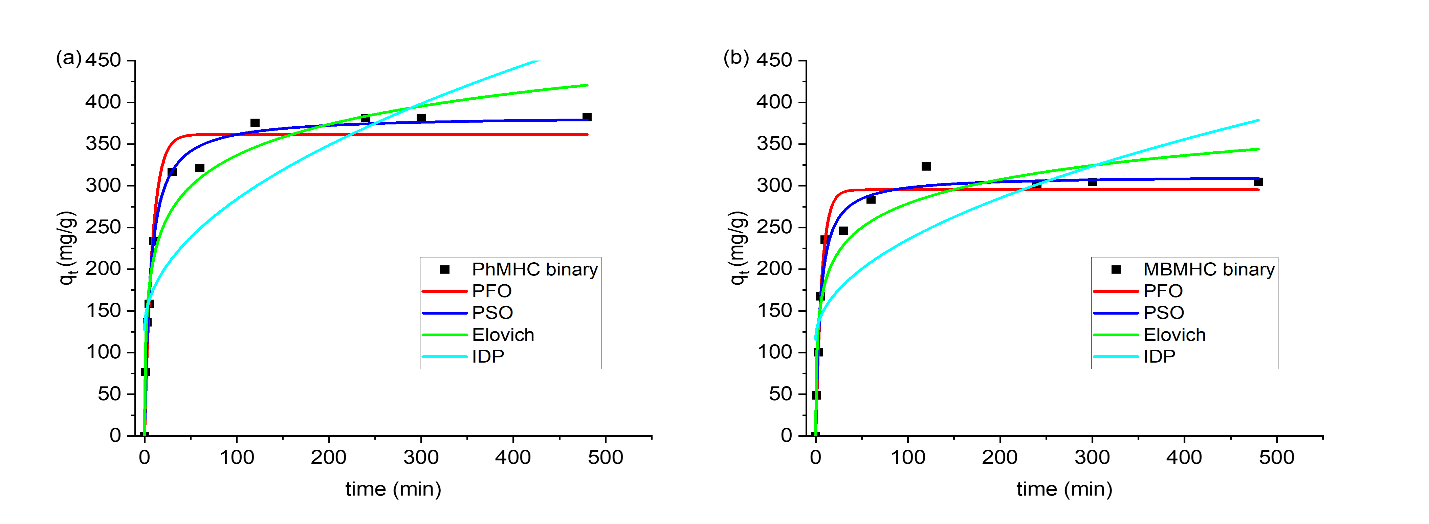

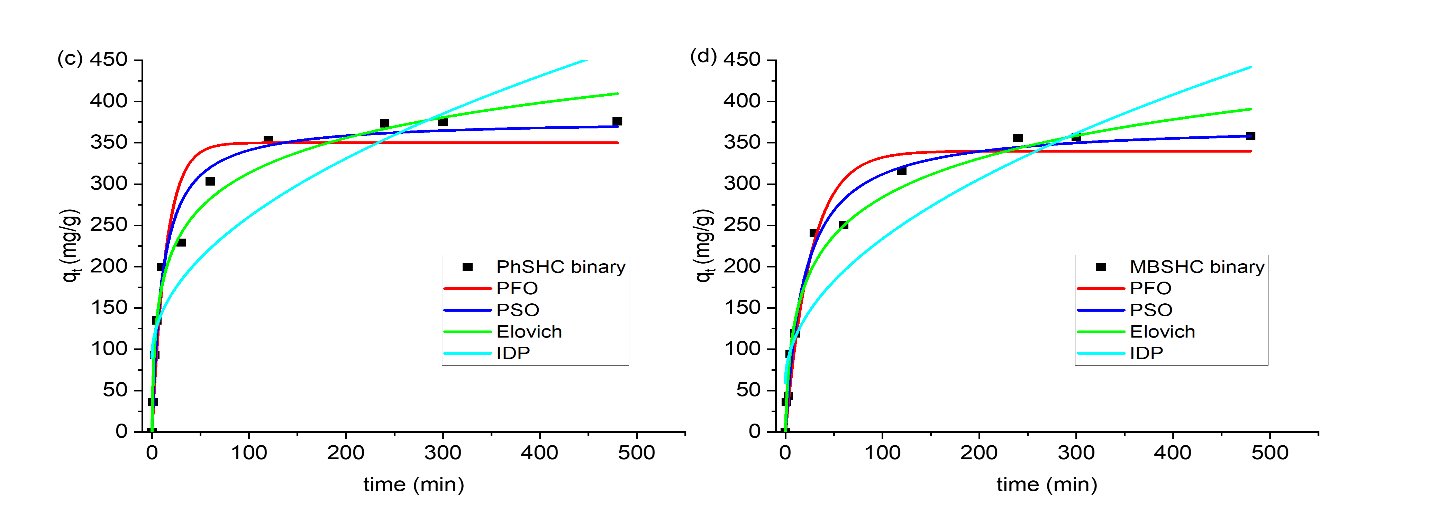

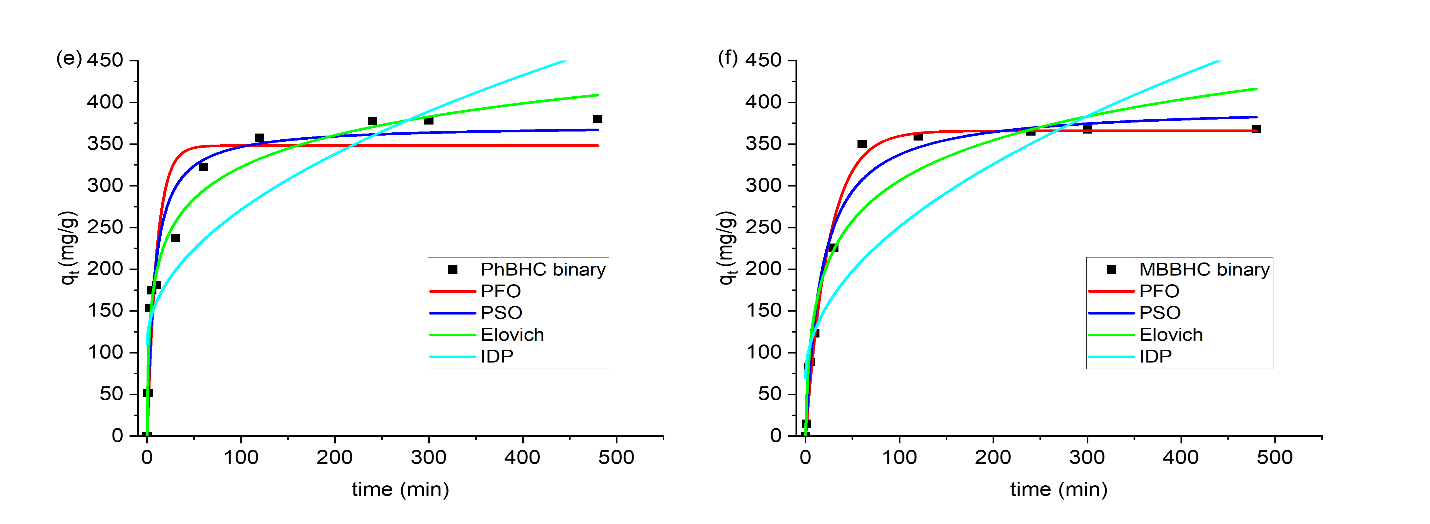


**Fig. S2:** Kinetic model for binary-adsorption (Ph-MB) onto MHC, SHC and BHC


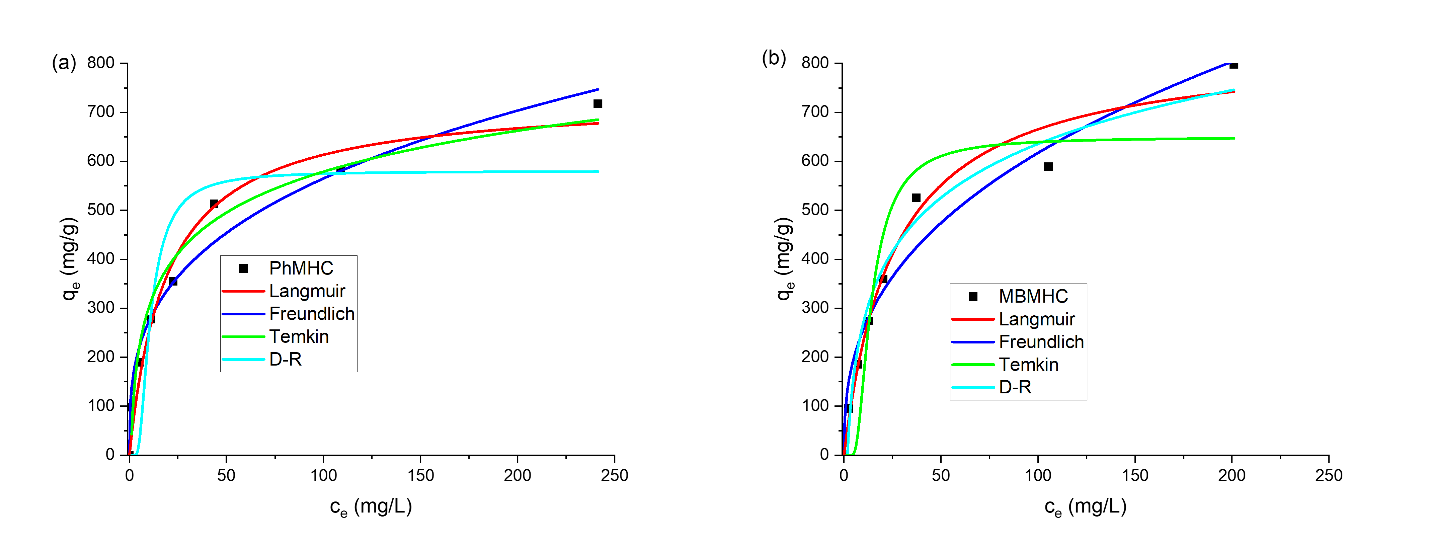

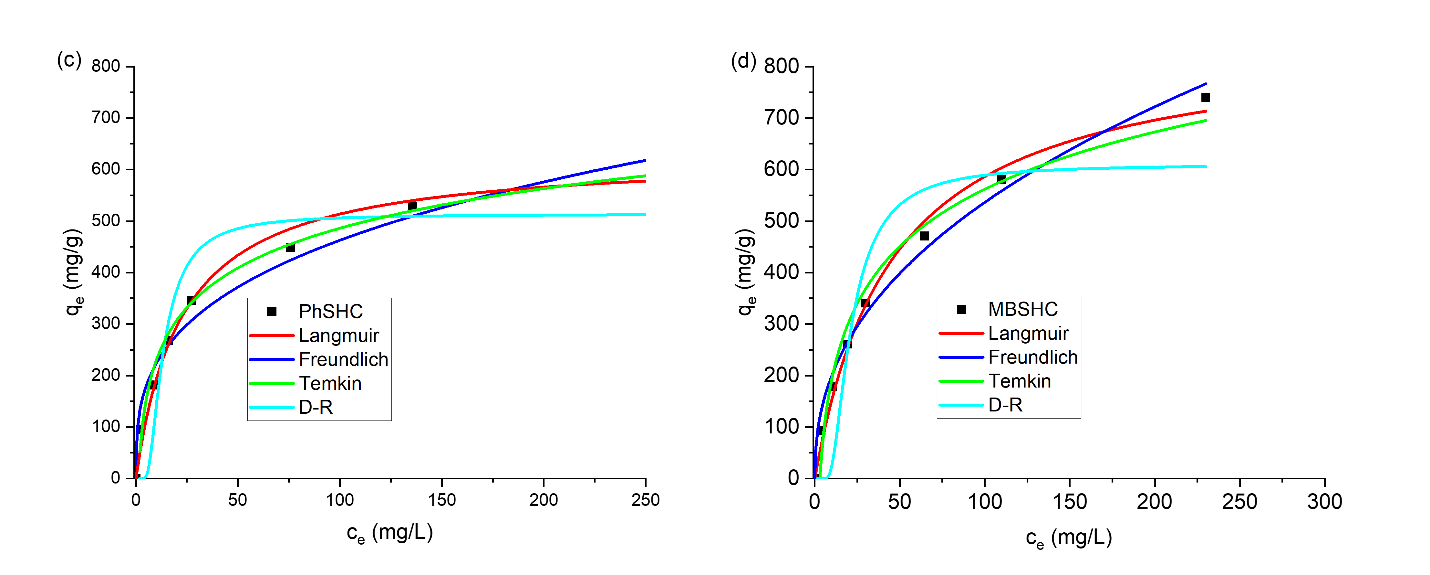

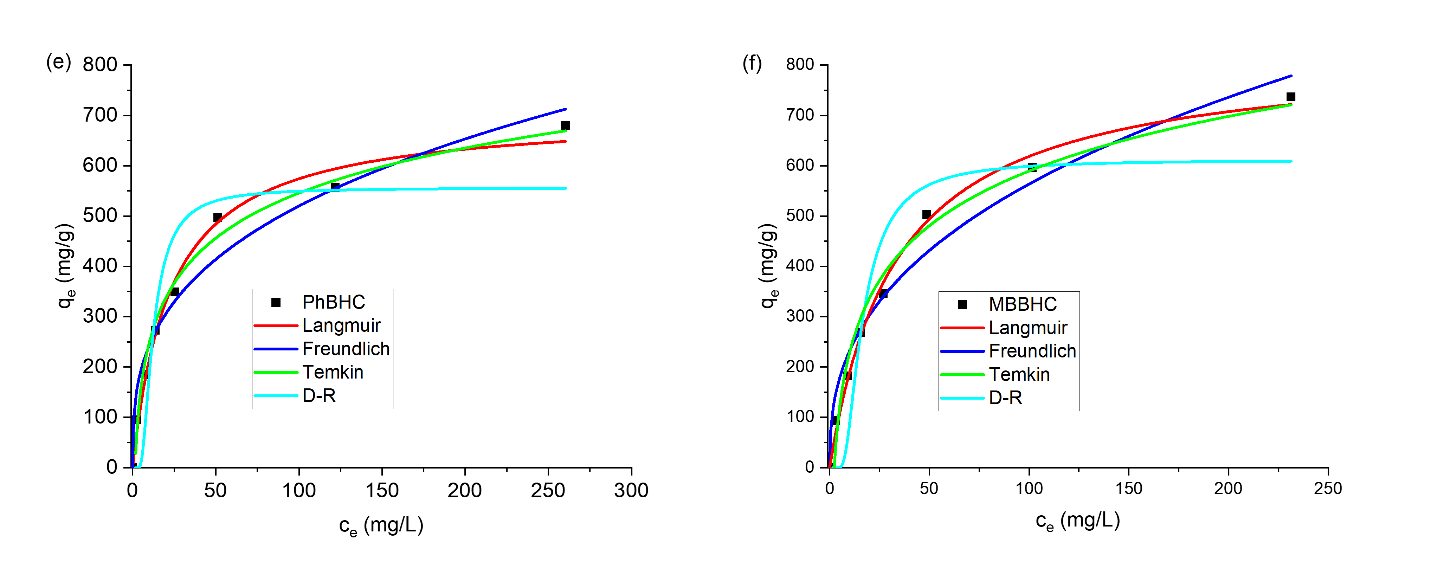


**Fig. S3:** Isotherm models of Ph-MB binary-adsorption system


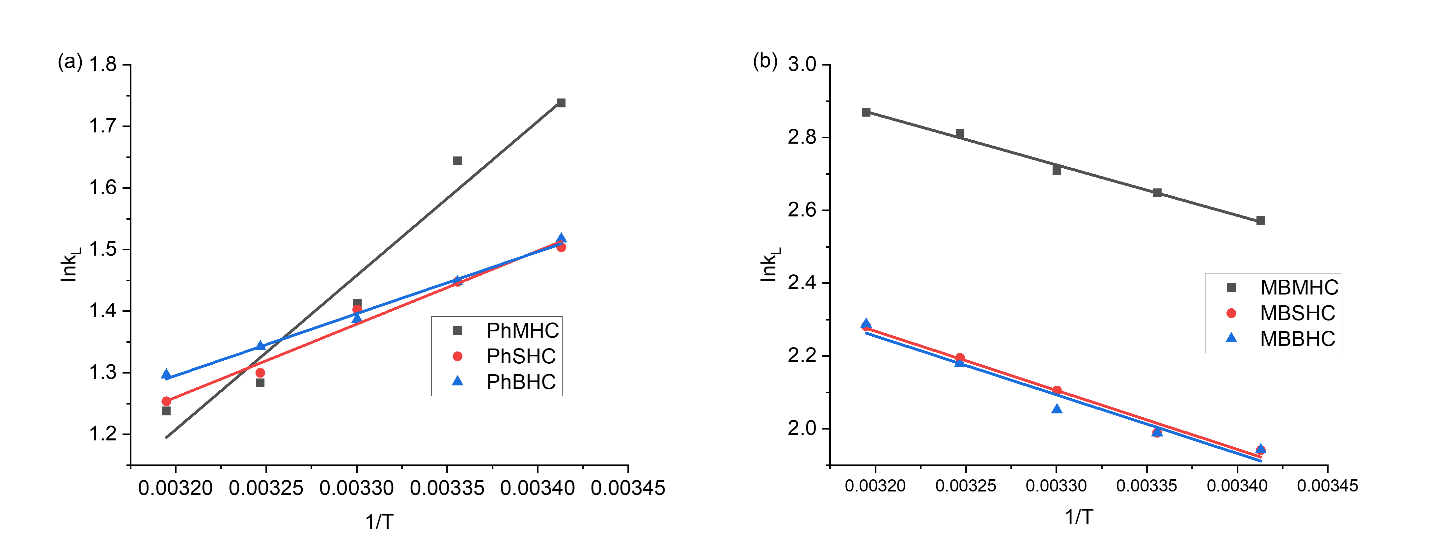


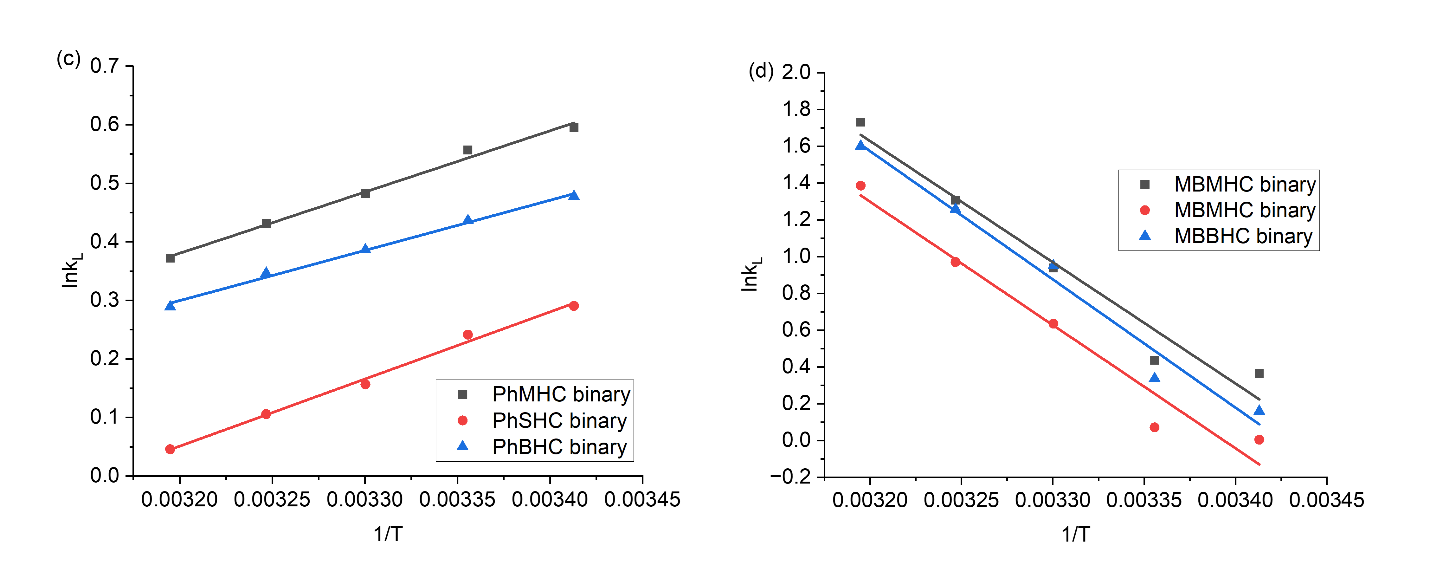


**Fig. S4:** Thermodynamic models of Ph-MB binary system

Table caption

**Table S1:** Kinetic models for binary Ph-MB adsorption parameters

**Table S2:** Isotherm models for binary Ph-MB adsorption parameters

**Table S1:** Kinetic models for binary Ph-MB adsorption parameters

| **Adsorbent** | **PFO** | | | **PSO** | | |
| --- | --- | --- | --- | --- | --- | --- |
|  | K_1_ (1/min) | q_e_ (mg/g) | R^2^ | K_2_ (g/mg/min) | q_e_ (mg/g) | R^2^ |
| PhMHC | 0.11 | 361.66 | 0.9653 | 0.00047 | 384.01 | 0.9908 |
| PhSHC | 0.06 | 349.98 | 0.9405 | 0.00024 | 377.88 | 0.9818 |
| PhBHC | 0.09 | 348.21 | 0.8956 | 0.00035 | 446.28 | 0.9549 |
| MBMHC | 0.15 | 295.26 | 0.9733 | 0.00066 | 312.09 | 0.9830 |
| MBSHC | 0.03 | 339.79 | 0.9682 | 0.00013 | 372.62 | 0.9894 |
| MBBHC | 0.04 | 365.89 | 0.9823 | 0.00014 | 396.32 | 0.9828 |
|  | Elovich |  |  | IDP |  |  |
|  | A | B | R^2^ | c | k | R^2^ |
| PhMHC | 282.22 | 0.018 | 0.9680 | 127.51 | 15.64 | 0.7166 |
| PhSHC | 100.00 | 0.016 | 0.9802 | 90.04 | 17.03 | 0.8038 |
| PhBHC | 190.83 | 0.018 | 0.9757 | 110.20 | 16.11 | 0.7807 |
| MBMHC | 336.24 | 0.024 | 0.9207 | 115.46 | 12.01 | 0.6196 |
| MBSHC | 242.40 | 0.014 | 0.9797 | 159.23 | 17.44 | 0.8511 |
| MBBHC | 253.28 | 0.014 | 0.9475 | 169.86 | 18.11 | 0.7787 |

**Table S2:** Isotherm models for binary Ph-MB adsorption parameters

| **Adsorbent** | **Langmuir** | | |  | **Freundlich** | | | |
| --- | --- | --- | --- | --- | --- | --- | --- | --- |
|  | K_L_ (L/mg) | q_m_ | R^2^ | K_f_ (mg/g)(L/mg)^1/n^  35.67  29.01  32.21  33.46  23.18  26.99 | | | n | R^2^ |
| PhMHC | 0.01 | 456.39 | 0.9763 |  |  |  | 2.45 | 0.9923 |
| PhSHC | 0.01 | 399.99 | 0.9424 |  |  |  | 2.38 | 0.9792 |
| PhBHC | 0.01 | 450.08 | 0.9814 |  |  |  | 2.38 | 0.9850 |
| MBMHC | 0.01 | 420.21 | 0.9794 |  |  |  | 2.48 | 0.9810 |
| MBSHC | 0.01 | 352.25 | 0.9462 |  |  |  | 3.15 | 0.9775 |
| MBBHC | 0.01 | 408.22 | 0.9895 |  |  |  | 2.34 | 0.9931 |
|  | **Temkin** |  |  | **D-R** | |  |  |  |
|  | A | B | R^2^ | q_m_  324.79  285.37  319.84  299.71  257.07  287.81 | | E | k | R^2^ |
| PhMHC | 0.17 | 88.38 | 0.9800 |  |  | 2.30 | 0.69 | 0.8324 |
| PhSHC | 0.15 | 80.00 | 0.9587 |  |  | 1.87 | 0.68 | 0.7956 |
| PhBHC | 0.14 | 90.83 | 0.9828 |  |  | 2.03 | 0.61 | 0.8465 |
| MBMHC | 0.15 | 85.11 | 0.9832 |  |  | 1.78 | 0.67 | 0.8333 |
| MBSHC | 0.12 | 74.29 | 0.9589 |  |  | 1.19 | 0.56 | 0.8057 |
| MBBHC | 0.12 | 83.60 | 0.9912 |  |  | 1.52 | 0.54 | 0.8753 |
